# Chemical and Structural Stability of CsPbX_3_ Nanorods during Postsynthetic Anion-Exchange: Implications for Optoelectronic Functionality

**DOI:** 10.1021/acsanm.3c05024

**Published:** 2024-01-20

**Authors:** Je-Ruei Wen, Anna Champ, Giselle Bauer, Matthew T. Sheldon

**Affiliations:** † Department of Chemistry, Texas A&M University, College Station, Texas 77843-3255, United States; ‡ Department of Materials Science and Engineering, Texas A&M University, College Station, Texas 77843-3255, United States

**Keywords:** lead halide perovskite, metastable, optoelectronic, nanorod, anion exchange, stability

## Abstract

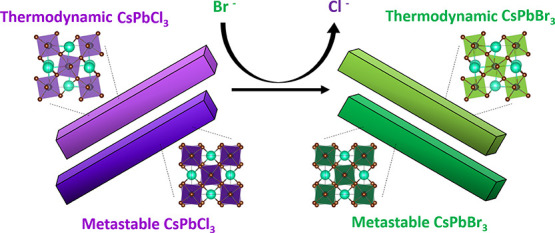

We examine halide anion-exchange reactions on CsPbX_3_ nanorods (NRs), and we identify reaction conditions that provide complete anion exchange while retaining both the highly quantum-confined 1-D morphology and metastable crystal lattice configurations that span a range between tetragonal structures and thermodynamically preferred orthorhombic structures. We find that the chemical stability of CsPbBr_3_ NRs is degraded by the presence of alkyl amines that etch CsPbBr_3_ and result in the formation of Cs_4_PbBr_6_ and 2-D bromoplumbates. Our study outlines strategies for maintaining metastable states of the soft lattices of perovskite nanocrystals undergoing exchange reactions, despite the thermodynamic driving force toward more stable lattice configurations during this disruptive chemical transformation. These strategies can be used to fine-tune the band gap of LHP-based nanostructures while preserving structure–property relationships that are contingent on metastable shapes and crystal configurations, aiding optoelectronic applications of these materials.

## 1 Introduction

Lead halide perovskites (LHPs) with the structure APbX_3_ (*X* = Cl, Br, I) are an emerging class of semiconductor materials that have gained intense attention due to their excellent optoelectronic properties. LHP colloidal nanocrystals (NCs) exhibit strong photoluminescence quantum yield (PLQY), have high defect tolerance, and possess bandgaps that are tunable across the entire visible spectral region.^1,2^ These promising features have prompted significant research into the use of LHP NCs for applications in photovoltaics,^3,4^ light-emitting devices,^5,6^ photodetectors,^7,8^ optical refrigeration,^9−11^ and photocatalysis.^12,13^

As with other quantum-confined materials, the bandgap of LHP NCs can be modulated by controlling particle size.^14^ However, an attractive feature of LHPs is that the bandgap can be tuned across the visible spectrum by manipulating halide composition, separately from the particle morphology, thus allowing for high color purity even for synthetic preparations that give relatively polydisperse particle size distributions. Halide stoichiometry is readily controlled by the molar ratio of halide precursors during crystal growth. Alternatively, halide stoichiometry can be altered postsynthetically via ion-exchange reactions by introducing supplementary halide species.^1,15,16^ Although modulating the halide ratio during the initial synthesis is often more straightforward, in some cases, the direct preparation of CsPbI_3_, CsPbCl_3_ or mixed halide LHP nanomaterials is not readily viable. In particular, syntheses of iodide perovskites are often more challenging, due to the higher precursor reactivity and instability, as well as the coexistence of multiple thermodynamically stable crystal phases corresponding to both desired and undesirable product structures.^17,18^ In this regard, ion-exchange becomes an alternative synthetic route to halide compositions with targeted optoelectronic properties.

Research on cation-exchange reactions involving colloidal metal chalcogenide NCs has been extensive. Interestingly, these reactions can preserve either thermodynamically stable or metastable lattice structures as well as complex NC morphology throughout the cation replacement process.^19,20^ This is made possible by the rigid anion frameworks, which provide kinetic stabilization of the overall structure under moderate reaction conditions.^21^ Additionally, these exchange reactions are typically irreversible under the same conditions, primarily due to the large thermodynamic driving force toward more favored products. To perform reverse cation-exchange reactions, it is necessary to alter the cations’ solvation energy, typically achieved by using different ligands or solvents. Without these modifications, a substantial excess concentration of the cations are required to surmount the energy barriers inherent in the reaction.^22^ In contrast, LHPs possess relatively soft crystal lattices with more similar crystal energies across all halide compositions, as well as similar halide ion solvation energies.^15^ These factors contribute to considerably lower energy barriers for both forward and reverse anion-exchange reactions, thereby facilitating a rapid exchange rate^15,23^ However, these unique attributes of the LHP lattices also raise questions about the ability of these materials to sustain a metastable structure throughout the exchange reactions.

Further, owing to the soft lattice and the highly mobile halide-bound, surface alkyl amine ligands, anion-exchange on LHPs can be conducted over a wide range of reaction conditions.^24^ A variety of halide species, such as metal halide solutions,^15^ benzoyl halides (Bz-X),^25^ trimethylsilyl halides (TMS-X),^26^ halohydrocarbons,^27−29^ or even the LHP NCs themselves^16^ can be utilized as halide sources for the postsynthetic exchange reactions. Usually, the reactions reach the end points in just a few minutes or less, even at room temperature. However, a complete exchange of halides is not always achievable, more often resulting in a thermodynamic mixture of halide compositions instead. Additionally, it is reported that LHP nanomaterials often lack structural stability throughout the course of anion-exchange reactions, particularly highly quantum-confined geometries like ultrathin nanowires, also converting between polymorphs.^30,31^ The poor stability is not fully understood, though it has been attributed to various factors, including chemical perturbation from excess ligands in the halide stock solutions as well as crystal disintegration and reconstruction during some halide-exchange processes.^32,33^

**Scheme 1:**
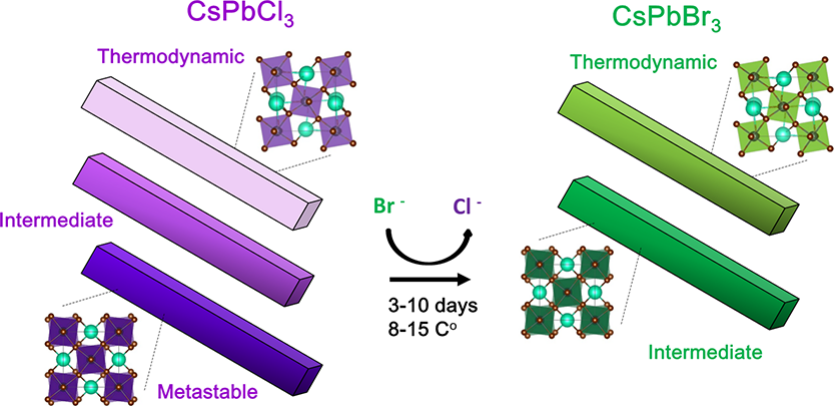
Quantum-Confined CsPbCl_3_ Nanorods (NRs) Can Be Prepared in Either an Orthorhombic Crystalline Phase—The Preferred Thermodynamic Lattice Configuration—or a Metastable Tetragonal Lattice Configuration (Left) Samples can also be prepared in an intermediate lattice configuration between these extremes that is also metastable. Synthetic conditions have been identified that allow for preservation of the overall NC morphology during anion-exchange reactions, resulting in quantum-confined CsPbBr_3_ NRs (right). The thermodynamic orthorhombic phase or metastable intermediate lattice configurations are preserved during the complete, stoichiometric anion exchange.

**Figure 1:**
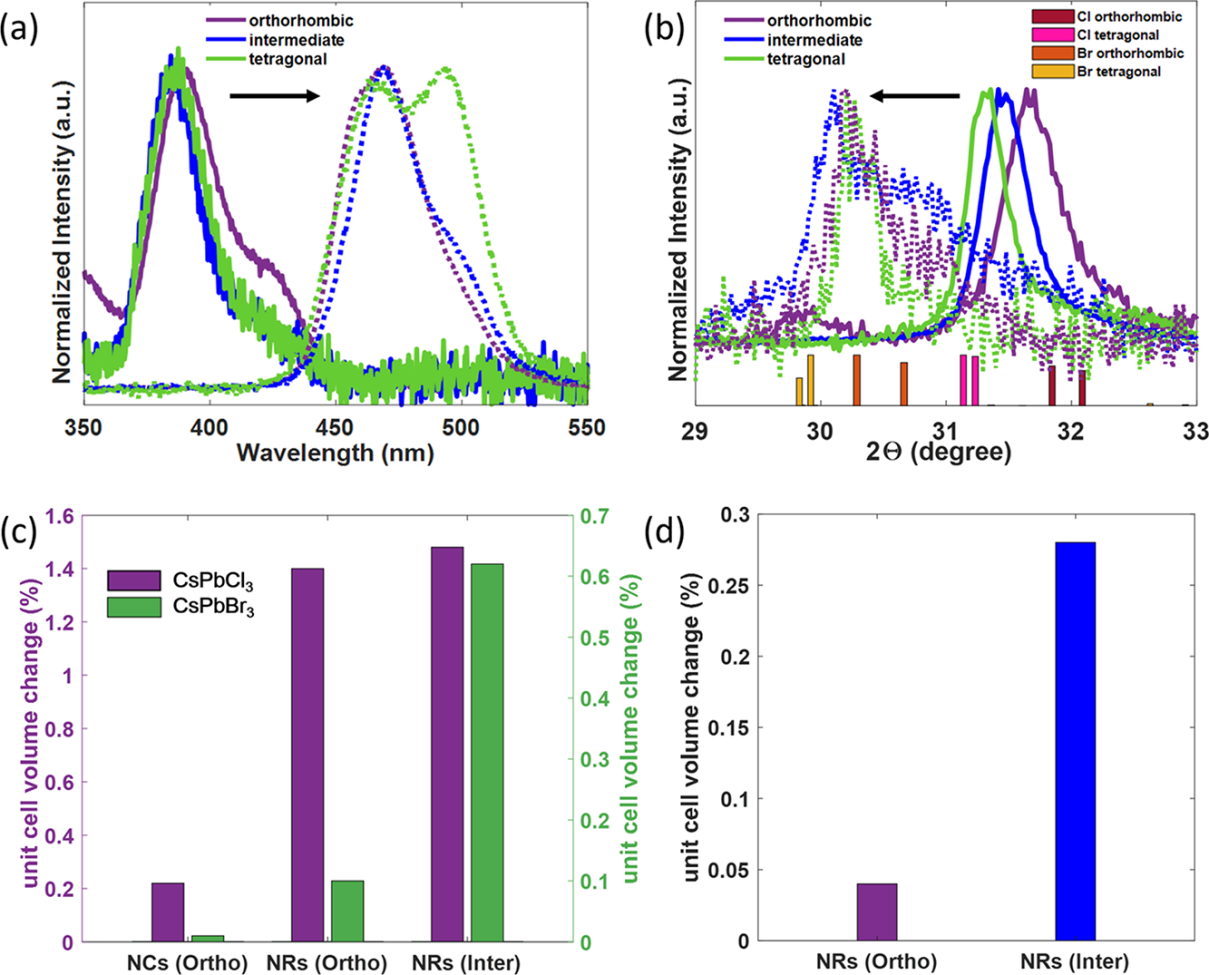
(a) UV–vis spectra and (b) XRD patterns of parent CsPbCl_3_ (solid) and corresponding Br-exchanged nanorods (dashed) in different crystal configurations. The exchange reactions are performed for 3, 4, and 9 days for the orthorhombic (Ortho), intermediate (Inter), and tetragonal samples, respectively. Change in the volume of unit cell as compared to bulk materials for (c) directly synthesized and (d) anion-exchanged CsPbBr_3_ NRs.

**Figure 2:**
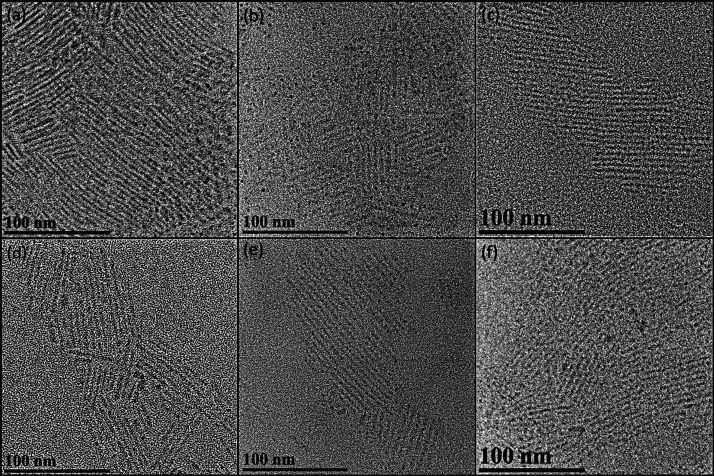
TEM images of (a–c) parent CsPbCl_3_ and (d–f) corresponding Br-exchanged nanorods. The samples are in (a,d) tetragonal, (b,e) intermediate, and (c,f) orthorhombic configurations.

Recently we developed a strategy for the kinetically controlled preparation of highly quantum-confined CsPbX_3_ nanorods (NRs) (Figures 1 and 2, Scheme 1).^17^ Based on synthetic conditions, the crystal lattice parameters of the NRs are continuously tunable between two extremes: the thermodynamically preferred orthorhombic structure and a metastable lattice geometry, which has been previously assigned to the I4/mcm tetragonal configuration (Figure S1). The metastable tetragonal CsPbX_3_ NRs clearly exhibit larger lattice volumes as compared to those of the conventional orthorhombic counterparts (Table S1). Furthermore, the metastable NRs are not indefinitely stable at room temperature and will slowly contract into the orthorhombic crystal structure over several months. In this study, the crystallographic and morphological stability of the highly quantum-confined, metastable CsPbX_3_ NRs over the course of anion-exchange reactions is explored. In addition, the chemical stability of the NRs against a variety of chemicals, including the starting precursors associated with their synthetic preparation, as well as several halide alternatives is investigated. We are surprised to identify reaction conditions that provide complete anion exchange while preserving the metastable crystal structure and nanorod morphology despite the clear preference of the material system to adopt the thermodynamically favored orthorhombic crystal lattice. This result has implications for fine-tuning the band gap of LHP-based nanostructures while preserving structure–property relationships that are contingent on metastable shapes and crystal configurations, such as the electron–phonon coupling efficiency,^2^ aiding optoelectronic applications for these materials.

## 2 Results and Discussion

In order to investigate the stability of CsPbX_3_ NRs during postsynthetic anion-exchange reactions, nanorod samples were prepared in either the orthorhombic or tetragonal lattice configurations, or in an intermediate configuration between these two extremes, using established methods.^17^ Before or after undergoing a cleaning and purification process, the NRs were subjected to various halide precursors, with effective reaction conditions being identified, as detailed further below. As displayed in Figures 1a,b and 2a–c, parent CsPbCl_3_ NRs with comparable size and quality were produced in three distinct crystal structures. The samples showed a monotonic downshift of X-ray diffraction (XRD) patterns from the orthorhombic to intermediate to tetragonal structures (Figure 1b, solid curves), suggesting continuous lattice expansion and distortion among the three samples of CsPbCl_3_ NRs. Note that only the scattering signal between 30 and 32 degrees exhibits a monotonic shift with crystal phase transition, which is particularly significant for distinguishing between the different structural phases. The complete XRD patterns that cover a broader angle range are provided in Figure S1 in the Supporting Information.

Further insight into the differences in crystal structures is obtained by closer analysis of the lattice parameters. XRD patterns of both the directly synthesized NRs (i.e., without exchange reactions performed) and conventional orthorhombic cuboid NCs were fitted using Pawley refinement (Figure S2). The fitted parameters were then compared to those of the bulk materials, as summarized in Tables 1 and S1. As demonstrated in Figure 1c, the cuboid NCs exhibited unit cell volumes quite similar to those of the bulk materials, while the directly synthesized NRs displayed larger values. This trend can be attributed to the relatively negative surface energy exhibited by more quantum-confined materials, leading to expanded lattice volumes.^34^ Additionally, the NRs in the intermediate crystal configuration displayed even larger unit cell volumes than those of the orthorhombic NRs. This was observed for the directly synthesized CsPbCl_3_ and CsPbBr_3_ NRs. These findings unambiguously indicate that NRs in the intermediate configuration are crystallographically distinct from their orthorhombic counterparts.

**Table 1:** Fitted Lattice Parameters of NCs and NRs as Compared with Bulk Materials

sample	*a* (%)	*b* (%)	*c* (%)	volume (%)
CsPbCl_3_ NCs (orthorhombic)	–0.20	–1.17	1.61	0.22
CsPbCl_3_ NRs (orthorhombic)	0.05	0.10	1.03	1.40
CsPbCl_3_ NRs (intermediate)	0.30	–0.10	1.27	1.48
CsPbBr_3_ NCs (orthorhombic)	0.01	–0.08	0.09	0.01
CsPbBr_3_ NRs (orthorhombic)	–0.13	0.16	0.01	0.04
CsPbBr_3_ NRs (intermediate)	–0.10	–0.03	0.33	0.20

To induce anion exchange, the crude nanorod solutions were injected into vials filled with metal halide powders and stirred at temperatures between 8 and 15 °C for several days prior to cleaning. After treatment with PbBr_2_ powders for 3, 4, and 9 days for the orthorhombic, intermediate, and tetragonal NRs respectively, the photoluminescence (PL) peaks red-shifted to approximately 470 nm (Figure 1a). This shift indicates complete halide exchange of chloride with bromide in the NRs. Despite our efforts, energy-dispersive X-ray spectroscopy (EDS) mapping was not able to distinguish quantitative differences in elemental composition between NRs that underwent complete anion exchange and those retaining mixed phases, as judged by the PL signal. Consequently, we have relied on the combination of PL emission wavelengths and XRD data as robust indicators of sample composition. Note that the red-side shoulder, and peak splitting, especially apparent in the rods after exchange, is likely due to trap emission, as discussed in depth in our previous report.^17^ Monodisperse NRs of all samples were observed by transmission electron microscopy (TEM), as illustrated in Figure 2d–f. The size and shape of these NRs were comparable to those of their corresponding parent NRs, clearly demonstrating the successful preservation of the highly quantum-confined 1-D morphology throughout the anion-exchange reactions. To further substantiate our findings, a statistical analysis of the nanorod size distribution was performed before and after the anion-exchange process, confirming the maintenance of the monosized distribution and morphological integrity (Table S3). This analysis, along with the consistent lattice parameters observed postexchange, provides compelling evidence of the phase purity and structural preservation of the CsPbX_3_ phase during the anion-exchange reaction. The exchange reaction also preserved the underlying crystallographic structure (Figure 1b) of the parent CsPbCl_3_ NRs in the orthorhombic and intermediate configurations. As indicated by the fitted lattice parameters (Figure 1d and Table S2), the crystal lattice of NRs with an intermediate configuration exhibited a larger lattice volume than did the orthorhombic NRs after exchange. These results confirm that a metastable crystal configuration can be sustained throughout anion-exchange reactions in the CsPbX_3_ system, even when the materials are highly quantum-confined and have significant shape anisotropy.

Nevertheless, the exchange reaction did not preserve the lattice configuration of parent CsPbCl_3_ NRs with the most pronounced tetragonal configuration, which is the least thermodynamically stable of the three lattice configurations that were analyzed. The tetragonal CsPbCl_3_ NRs were transformed to orthorhombic CsPbBr_3_ NRs, instead of preserving the original tetragonal crystal configuration (Figure 1b). This behavior may be attributed to a spontaneous structural transition into a more thermodynamically stable state over an extended reaction period. Additionally, a degradation of the crystallinity throughout the exchange reaction could contribute to this outcome. A decrease in crystallinity is suggested by the broadening and emergence of shoulders in the XRD pattern and the PL spectrum. Interestingly, despite these changes, the highly quantum-confined nanorod shape was remarkably well-preserved, as shown in Figure 2f.

Given that LHPs lack the rigid frameworks characteristic of metal chalcogenides and are widely recognized for their soft crystal lattices, one might expect some strain-induced lattice reorganization, or even collapse, during the halide-exchange processes. However, the preservation of the highly quantum-confined nanostructure and metastable crystal configuration suggests that the PbX_3_^–^ sublattice is more resilient than initially anticipated. The energy barrier for lattice distortion—i.e., conversion from a metastable state like the tetragonal or intermediate geometry into the orthorhombic structure—is apparently substantial enough to maintain the integrity of the nanomaterials. Moreover, our observations suggest that the exchange reactions demonstrated in this work likely follow a halide vacancy-assisted diffusion route,^15^ rather than proceeding through a process of disintegration and subsequent reconstruction.

In addition to studies of parent CsPbCl_3_ NRs, anion-exchange was also tested on tetragonal CsPbBr_3_ NRs by introducing PbCl_2_ and PbI_2_ powders. It was noticed that the Cl-exchange reactions took much longer time, roughly 3 weeks to reach the complete halide-exchange product (Figure S4a). While the Cl-exchanged products retained the highly quantum-confined 1-D morphology and good optical quality (Figure S4a,d), the NRs were crystallographically degraded. Further, the parent metastable tetragonal architecture was not retained (Figure S4b). On the other hand, attempts of treating CsPbBr_3_ NRs with PbI_2_ were less successful. The samples were completely degraded, turning into nonfluorescent, milky white solutions after a few days before reaching the full exchange product. Evidently, the unsuccessful exchange is influenced by additional factors and not only the spontaneous structural conversion which is dictated by thermodynamics. A thorough understanding of the causes of this degradation is therefore necessary.

**Figure 3:**
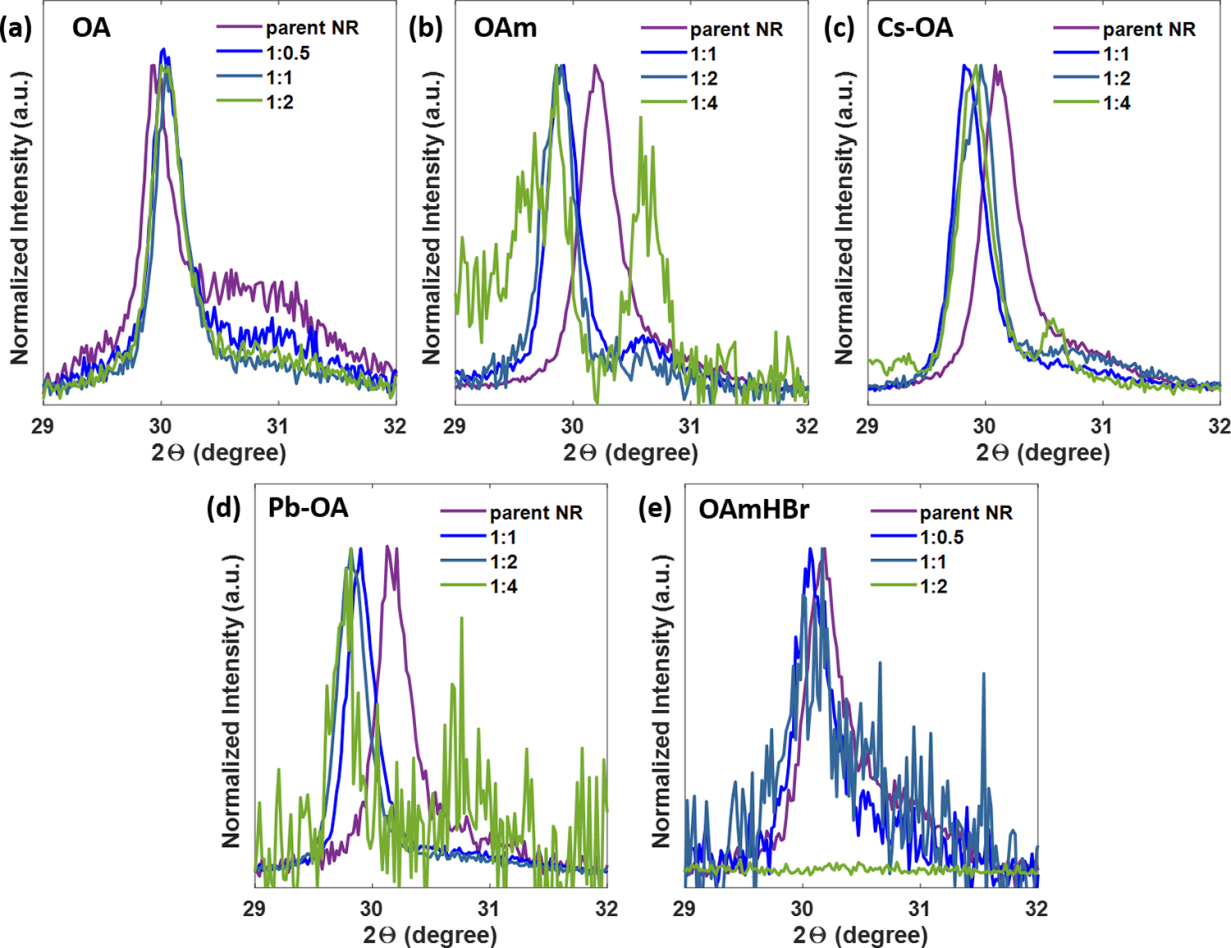
XRD patterns of CsPbBr_3_ NRs treated with additional (a) OA, (b) OAm, (c) Cs-OA, (d) Pb-OA, and (e) OAmHBr. The ratios (colored traces) indicate the molar ratios between the amount of that chemical in the crude solution and the supplemental amount added.

To investigate the chemical degradation, solutions of tetragonal CsPbBr_3_ NRs were individually treated with additional oleic acid (OA), oleylamine (OAm), lead oleate (Pb-OA), cesium oleate (Cs-OA), and oleylammonium bromide (OAmHBr). Each of these chemical species are used during the synthesis of parent NRs,^17^ so they are present in a complex equilibrium in the purified products. Perturbation of any of the concentrations of these species during an exchange reaction was, therefore, hypothesized to be the cause of the chemical degradation. As revealed in Figures 3a and S5a, additional OA had minimal effect on the NRs as only trivial changes could be seen in the XRD and UV–vis spectroscopy, likely because the system already contains a high concentration of OA based on the initial synthetic preparation. In contrast, the NRs demonstrated a pronounced sensitivity to supplementary OAm, as shown in Figures 3b and S5b. With increasing the OAm concentration, the XRD pattern shifted to lower angles, while the absorption edge and the PL peak shifted to higher energy. The shift of the XRD pattern toward smaller angles may be attributed to either the stabilization of a more tetragonal phase, which typically stops shifting at around 29.8°—the theoretical diffraction position for the (0 0 4) plane—or to a negative surface energy indicative of lattice expansion. These observations are consistent with reported phenomena in which lattice expansion has been linked to shifts in diffraction patterns. In fact, with an elevated concentration of additional OAm, the NRs did not preserve the perovskite crystal structure. Instead, they transitioned into the lead-deficient phase, Cs_4_PbBr_6_, as evidenced by the emergence of a diffraction reflection around 30.6° in Figure 3b. This behavior can be attributed to the extraction of bromide ions from the NRs by the generated oleylammonium (eqs 1and 2), a chemical process frequently observed in similar systems where PbBr_2_ is extracted while leaving the Cs^+^ substructure.^37,38^ Similar to OAm, when the NR solution gained supplemental Cs-OA, the system became increasingly deficient in PbBr_2_, and ultimately yielded Cs_4_PbBr_6_ (Figures 3c and S5c) (eq 3). From these observations, it can be inferred that the blue-shift in the UV–vis spectra was due to etching of the perovskite NRs:
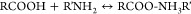
1
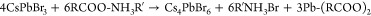
2
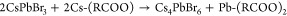
3

Conversely, when incorporating Pb-OA stock solutions, one might expect either no reaction or the transformation to CsPb_2_Br_5_, given the lead- and bromide-rich environment (eq 4).^35^ However, against expectations, the addition of Pb-OA led to an evident dissolution of the products, as shown in Figure S6d. This was further confirmed by the blue-shift observed in the UV–vis spectra, as well as a decrease in the nanorod diameters with higher Pb-OA concentrations (Figures S5d, S7 and Table S3). When the added Pb-OA was quadrupled, the formation of lead-deficient products was even observed (Figure 3d). Thus, it was hypothesized that the lead oleate may compete with the perovskite solids for bromide ions, extracting the bromides and forming higher order coordination complexes in the liquid phase (eq 5). This process could be considered the reverse reaction of perovskite growth^36^:
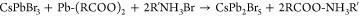
4
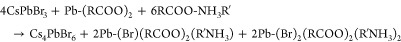
5

In the case of the OAmHBr, it was hypothesized that flooding the system with halides would push the equilibrium toward the more thermodynamically stable state, namely, orthorhombic CsPbBr_3_. However, contrary to expectations, this chemical did not induce the expected change (Figure 3e). Instead, the solution transitioned from a light greenish-yellow hue to a milky white, resulting in a significant increase in precipitate (Figure S6e). As observed in Figure S5e, there are blue-shifted UV–vis spectra at higher OAmHBr concentrations. Ultimately, the absorption edge corresponding to CsPbBr_3_ vanished, while a robust absorption peak characteristic of Cs_4_PbBr_6_, occurring around 310 nm, emerged, accompanied by a pronounced scattering background due to the presence of larger particles. Subsequent XRD analysis confirmed the degradation of perovskites and the formation of highly ordered materials, as indicated by the low angle region (Figure S8). These observations underscored the fact that OAmHBr converted CsPbBr_3_ NRs to Cs_4_PbBr_6_ and potentially, 2-D bromoplumbates, expressed as (R’NH_3_^+^)_n_(PbBr_2+n_^n–^).^37,39^ This suggests that additional OAmHBr extracted lead bromide from the perovskite crystals, generating 2-D bromoplumbates and leaving lead-deficient products (eq 6). From these observations, it was concluded that the perovskite NRs exhibited poor chemical stability when exposed to high concentrations of most of the starting precursors:
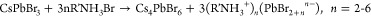
6

The study presented the above offers insight into the chemical degradation of highly quantum-confined CsPbX_3_ nanomaterials during anion-exchange reactions, including when CsPbBr_3_ NRs are mixed with PbI_2_ powders. Besides promoting halide exchange with the CsPbBr_3_ NRs or free OAmHBr in solution, the PbI_2_ powder likely undergoes dissolution by free OA and OAm present in the system. This process results in the generation of Pb-OA and OAmHI, which ultimately contribute to unwanted conversion reactions. Therefore, preventing the formation of Pb-OA or OAmHX would be necessary to ensure successful complete anion-exchange reactions.

Given that PbX_2_, Pb-OA, or OAmHX alone can convert perovskite NRs into different lead halide-based species, we sought to identify conditions that may provide an equilibrium between these reactive species in order to reduce perovskite degradation during anion-exchange reactions (eq 7). To do this, we combined the NRs with a variety of mixtures of OAmHBr and Pb-OA. However, each attempt resulted in degradation of the perovskites (Figure S9). The combination of OAmHBr and Pb-OA resulted in the production of oleylammonium oleate (eq 8). At high concentrations, the chemical destabilizes the perovskite structure, as previously discussed in relation to eq 2:
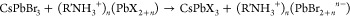
7
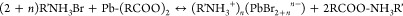
8

To identify a strategy that avoided the degradation of NRs, other halide-containing chemicals were explored as alternatives for OAmHBr. It has been reported that Bz-X and TMS-X are good candidates for anion-exchange reactions on LHP NCs.^25,26^ Benzoyl halides react with both carboxylic acids and amines to produce HX (eqs 9 and 10).^25^ However, HX was not stable in the nonpolar system and thus complexed with OAm to make OAmHX (eq 11). Then, the active reactant was OAmHX, which is no different from what had been explored above, such that too much Bz-X leads to degradation of perovskite NRs as well (Figure S10):
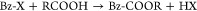
9
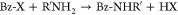
10
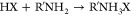
11

Regarding TMS-X, the Gamelin group has demonstrated that iodide or bromide can selectively replace bromide or chloride, respectively, in the perovskite structure (eq 12).^26^ However, a concurrent reaction between TMS-X and ammonium oleate can also take place, which, in turn, leads to the production of OAmHX (eq 13). The competition between these two reaction routes made it not possible to reach a complete anion-exchange without damaging the NRs (Figures S11 and S12). Therefore, both Bz-X and TMS-X proved unsuitable for the anion-exchange reaction in this system, which retained high concentrations of ligands even after cleaning.
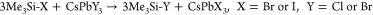
12
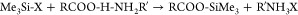
13

It appears that the formation of OAmHX was an unavoidable outcome with all of these halide carriers. As such, halide sources that may inhibit the formation of 2-D bromoplumbates are preferable. To this end, we explored the use of secondary and tertiary ammonium bromides, surmising that steric effects may interfere with the formation of bromoplumbates. However, as demonstrated in Figure S13, we observed clear degradation of the perovskite NRs and the emergence of 2-D structures even when treated with dihexylammonium bromide or trioctylammonium bromide. It seems that the harmful effects on LHPs caused by excess alkyl ammonium salts cannot be avoided before achieving complete halide exchange.

In light of the above unsuccessful attempts, halide species that undergo metathesis with the perovskites (direct exchange) or solubilized OAmHX already in solution (indirect exchange) may be preferable. To this end, alkyl halides, such as haloalkanes or metal halide powders with low solubility in this system, would seem to be ideal halide candidates. However, it is important to note that these compounds should also exhibit reasonable exchange reaction rates to prevent spontaneous structural conversion. Further attempts using ZnX_2_, CuX_2_, NaX, and KX were thus performed. The reaction rates were faster using ZnBr_2_ and CuBr_2_, but nonetheless, the NRs were completely degraded in a few minutes when mixed with ZnBr_2_ and within 2 h when mixed with CuBr_2_. We suspect these salts may have much higher solubility in the system and that the cations promote undesired disintegration of LHP crystals.^33^ On the other hand, sodium and potassium salts that are less soluble, not surprisingly, resulted in even slower exchange reactions. The sodium and potassium salts allowed for complete halide exchange of the NRs that preserved their morphology and optical quality. However, sodium and potassium salts did not preserve the tetragonal lattice structure of the parent NRs after exchange. We find that preservation of the highly quantum-confined shape is quite viable with anion-exchange reactions as long as the equilibrium of ligands between the solution and nanorod surface is not significantly perturbed, whereas maintaining the underlying crystallinity and metastable crystal configuration requires more special care.

## 3 Conclusions

In this investigation of CsPbX_3_ NRs, we not only explored anion-exchange reactions but also established reaction conditions that effectively preserved the unique quantum-confined one-dimensional morphology of the NRs. Crucially, we also succeeded in maintaining the metastable intermediate crystal structure, a surprising and significant outcome given the tendency of these materials to revert to a more stable orthorhombic lattice. Our scrutiny into the chemical stability of tetragonal CsPbBr_3_ NRs under various conditions revealed a susceptibility to degradation by numerous chemicals, including those used as synthesis precursors. Notably, while oleic acid exerted minimal influence, the introduction of alkyl amines, whether added directly or generated in situ, led to the etching of CsPbBr_3_ and the formation of secondary phases such as Cs_4_PbBr_6_ and two-dimensional bromoplumbates. The quest for optimal halide carriers that promote efficient exchange without damaging the lattice of LHPs continues. Yet, the consistent preservation of intermediate structures postexchange in our study underscores the potential to maintain ‘soft’ lattice structures through halide-exchange processes, even in less stable, metastable states. This achievement opens new avenues for the precise band gap engineering of LHP-based nanostructures that can aid optoelectronic functionality while retaining structure–property relationships critical to their metastable forms and crystal configurations.

## Supporting Information Available

Materials preparation and characterization including XRD patterns, tabulated structural data, and TEMs; and additional control experiments (PDF)
