# Supplementary material for: Chemical and Structural Stability of CsPbX3 Nanorods during Postsynthetic Anion-Exchange: Implications for Optoelectronic Functionality
Source: ACS Appl Nano Mater. 2024 Jan 20;7(3):3024–31. doi: 10.1021/acsanm.3c05024 (PMC10862380; doi:10.1021/acsanm.3c05024)
Supplement: Supplementary file 1 — an3c05024_si_001.pdf [file an3c05024_si_001.pdf]

Supporting Information

Chemical and Structural Stability of CsPbX3 Nanorods During Post-Synthetic Anion-Exchange: Implications for Optoelectronic Functionality

Je-Ruei Wen[1], Anna Champ[1], Giselle Bauer[1], Matthew T. Sheldon[1,2]*

[1]Department of Chemistry, Texas A&M University, College Station Texas, 77843-3255, USA
[2]Department of Materials Science & Engineering, Texas A&M University, College Station Texas, 77843-3255, USA

Corresponding Author
Matthew T. Sheldon − orcid.org/0000-0002-4940-7966; Email: sheldonm@tamu.edu

Materials. Cesium carbonate (Cs_2CO_3, 99.9%), lead oxide (PbO, 99%), oleic acid (OA, 90%), oleylamine (OAm, 70%), 1-octadecene (ODE, 90%), trioctylphosphine (TOP, 97%), dihexylamine (DHAm, 97%), trioctylamine (TOAm, 98%), benzoyl chloride (Bz-Cl, 99%), trimethylsilyl iodide (TMS-I, 97%), lead iodide (PbI_2, 99.999%), cupric bromide ($CuBr_2$, 99%), potassium bromide (KBr, 99+%), potassium iodide (KI, 99+%), toluene (99.8%) and hexane (95%) were received from MillliporeSigma. Lead bromide ($PbBr_2$, 98+%), lead chloride ($PbCl_2$, 99.999%) and zinc bromide ($ZnBr_2$, 99.9%) were purchased from Alfa Aesar. Sodium bromide (NaBr) was received from Johnson Matthey. Hydrobromic acid (HBr, 48%) was purchased from VWR Chemicals BDH. Hydrochloric acid (HCl, 36.5%-36.8%) was received from Macron Fine Chemicals. OA and OAm were dried with molecular sieves under Ar environment before use. NaBr and KBr were dried at 80 °C overnight then stored under Ar environment. Other chemicals were used as received.

Preparation of cesium oleate (Cs-OA) stock solution. 0.2 g Cs_2CO_3 was reacted with 0.6 mL OA in 10 mL ODE at 120 °C under vacuum for 1 hour. Once the chemical was dissolved, additional 1.6 mL OA was added under Ar atmosphere, and the solution was allowed to cool down to room temperature, transferred to an Ar-filled vial for storage.

Preparation of lead oleate (Pb-OA) stock solution. 0.185 g PbO was loaded into a 25 mL flask along with 4.3 mL ODE and 0.64 mL OA, dried at 125 °C under vacuum for 1.5 hour until the chemical was fully solubilized.

Preparation of oleylammonium bromide (OAmHBr) and oleylammonium chloride (OAmHCl). 15 mL undried OAm was added in 30 mL acetonitrile under vigorous stirring in an Ar-filled covered beaker. 4 mL for HBr or 3 mL for HCl was then injected drop by drop into the solution, which was cooled in a water bath. The solution was stirred for 4 hours at room temperature. The precipitate was then purified with diethyl ether and hexane several times, dried under vacuum at 60 °C overnight, and stored in glovebox for future use.

Preparation of dihexylammonium bromide (DHAmHBr). In a beaker containing 20 mL acetonitrile

and 8 mL DHAm, 5 mL HBr was added dropwise with water bath cooling. The mixture was stirred at room temperature for 3 hours. The precipitate was then purified with acetonitrile and hexane several times, dried under vacuum overnight, and stored in glovebox for future use.

Preparation of trioctylammonium bromide (TOAmHBr). 4.5 mL HBr was added drop by drop into 10 mL of TOAm under vigorous stirring. The cloudy mixture was stirred for another 2 hours, filtered and rinsed with D.I. water several times. The products were dried at 80 °C overnight, stored in glovebox for future use.

Preparation of benzoyl iodide (Bz-I). 1.4 mL Bz-Cl was added to an Ar-filled vial containing 3 g KI. The mixture was stirred at around 60 °C for one day covered with aluminum foil. 3 mL dried ODE was added to the vial, and the supernatant was collected with a 0.2 μm syringe filter, stored in glovebox for future use.

Preparation of PbX_2 stock solutions. 2.5 mL ODE and 0.2-0.4 mmol PbX_2 were loaded into a 2-neck flask and dried under vacuum at 120 °C. 0.7 mL OA and 0.7 mL OAm were added under Ar atmosphere and the solution was stirred until complete dissolution of the salt. For $PbCl_2$, an additional 1 mL TOP was added to the flask. The resulting concentrated stock solution was cooled to room temperature and transferred to an Ar-filled vial for storage.

Synthesis of tetragonal $CsPbBr_3$ nanorods. 5 mL ODE was loaded into a round button flask and dried under vacuum at 120 °C. It was al-lowed to cool down to 80 °C, and 0.3 mL of Pb-OA stock solution was injected. To prepare the Cs feedstock, 0.3 mL Cs-OA stock solution was mixed with 0.55 mL OA, 0.25 mL OAm, and 0.27 mL ODE. Separately, 0.085 g OAmHBr was mixed with 0.7 mL toluene and 0.4 mL ODE. 0.9 mL of each of the two feedstock solutions were individually injected into the flask at a rate of 3.6 mL/hr. The samples was purified by centrifugation at 10000 rcf for 20 min. The supernatant was discarded, and the precipitate was redispersed in hexane and centrifuged at 2000 rcf for another 10 min. The final supernatant was collected for future analysis.

Synthesis of $CsPbCl_3$ nanorods. $CsPbCl_3$ nanorods in different crystal phases were synthesized in ways similar to that of tetragonal $CsPbBr_3$ nanorods above. 0.75 g OAmHCl was used instead, and Cs feedstock solution was prepared with required amounts of OA and OAm for different crystal phases.

Post-synthetic treatment on $CsPbBr_3$ nanorods. Cooled crude solutions of $CsPbBr_3$ nanorods were transferred to Ar-filled vials. Desired amount of chemicals were then injected drop by drop under vigorous stirring at room temperature, and the mixtures were let stirring for another 1~2 hours before cleaning.

Post-synthetic treatment using metal halide powders. To an Ar-filled vial containing 0.5 g metal halide powder (PbI_2, $PbCl_2$, $PbBr_2$, $ZnBr_2$, $CuBr_2$, NaBr or KBr), 2 mL cooled crude solution of $CsPbBr_3$ or $CsPbCl_3$ nanorods was injected. The mixture was vigorously stirred in a water bath kept at 8-12 °C for some time, ranging from tens of minutes to dozens of days before cleaning.

Characterization. XRD data was acquired on a BRUKER D8-Focus Bragg-Brentano X-ray Powder Diffractometer equipped with Cu K-α radiation source. UV-Vis spectra were measured on an Ocean Optics Flame-S-UV-Vis Spectrometer with an Ocean Optics DH-200-Bal deuterium and halogen lamp

light source. TEM images were taken on a FEI Tecnai G2 F20 ST FE-TEM operated at 200 kV equipped with Gatan CCD camera.

Lattice parameter analysis. The lattice parameters of the nanorods and conventional nanocrystals were analyzed using the software package GSAS-II. The XRD patterns were fitted to the orthorhombic phases ($CsPbCl_3$: ICSD#243734; $CsPbBr_3$: ICSD#244752) with the Pawley refinement. The tetragonal $CsPbBr_3$ nanorods were fitted to the tetragonal phase (mp-1014168).

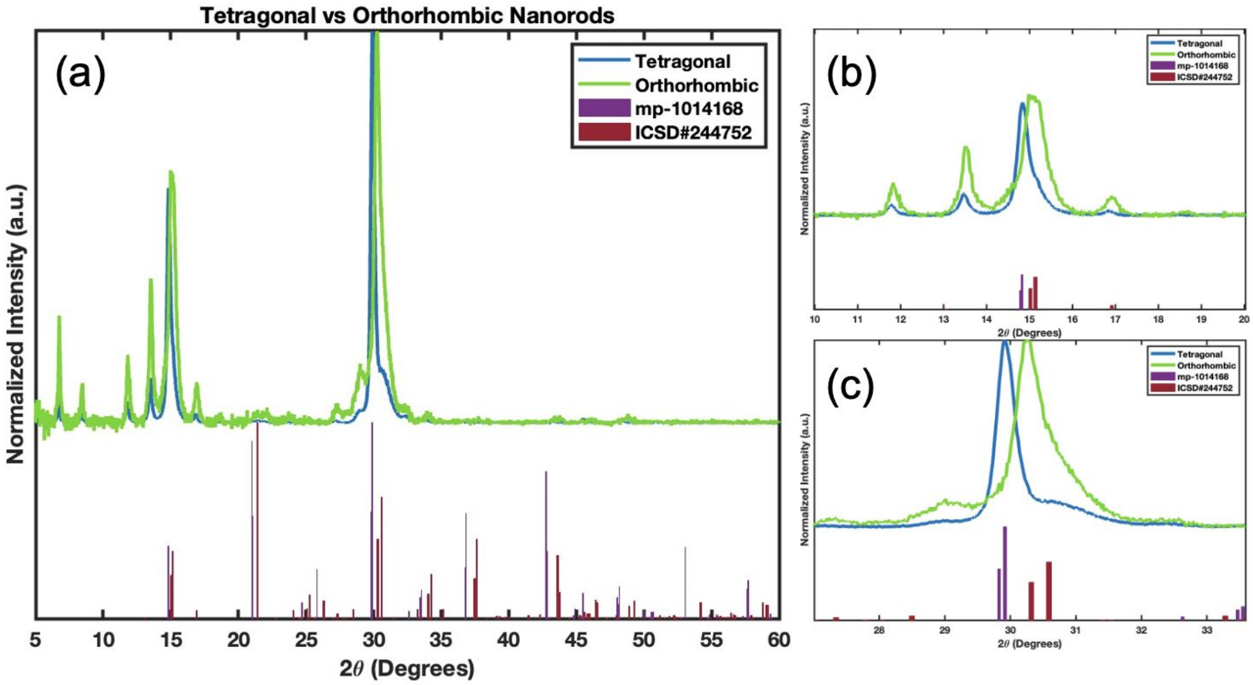

Figure S1. XRD patterns of tetragonal and orthorhombic $CsPbBr_3$ nanorods (a). Close-ups on the reflections at 15 degrees (b) and 30 degrees (c). The card files for the corresponding structures (mp-1014168; ICSD#244752) are provided. Note that the peaks below 15 degrees correspond to stacked nanorods that order during the preparation of the XRD plate.

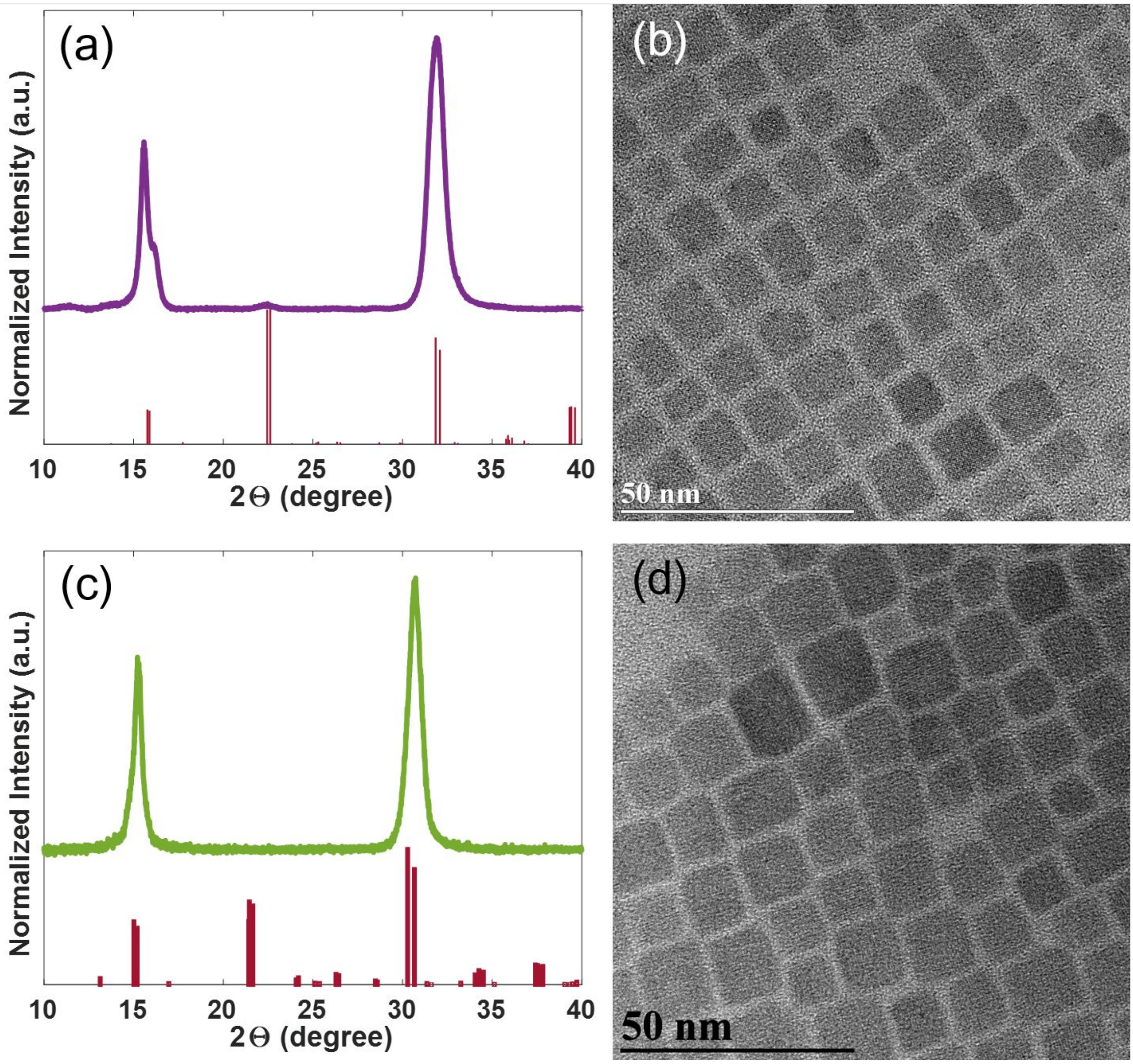

Figure S2. XRD and TEM results of (a,b) $CsPbCl_3$ and (c,d) $CsPbBr_3$ orthorhombic NCs. The card files for the corresponding structures (ICSD#243734; ICSD#244752) are provided.

Table S1. Fitted lattice parameters of NCs and directly synthesized NRs.

sample	a	b	c	volume	Rwp (%)
orthorhombic $CsPbCl_3$ bulk[#]	7.901931	7.899278	11.247778	702.080	--
$CsPbCl_3$ NCs (orthorhombic)	7.88651	7.80680	11.4284	703.628	54.35
$CsPbCl_3$ NRs (orthorhombic)	7.9056	7.90709	11.36356	711.938	57.525
$CsPbCl_3$ NRs (intermediate)	7.92596	7.89118	11.39109	712.457	48.74
orthorhombic $CsPbBr_3$ bulk*	8.2105	8.2829	11.8039	802.7448	--
$CsPbBr_3$ NCs (orthorhombic)	8.21093	8.27608	11.81396	802.809	27.67
$CsPbBr_3$ NRs (orthorhombic)	8.21117	8.28292	11.81502	803.569	69.266
$CsPbBr_3$ NRs (intermediate)	8.21337	8.29794	11.85098	807.692	72.673
$CsPbBr_3$ NRs (tetragonal)	8.43024	8.43024	12.02621	854.690	43.419
tetragonal $CsPbBr_3$ bulk[+]	8.447114	8.447114	11.981346	854.9137	--

[#]ICSD#243734; [*]ICSD#244752; [+]mp-1014168.

Table S2. Fitted lattice parameters of anion-exchanged nanorods

sample	a	b	c	volume	Rwp (%)
$CsPbBr_3$ NRs (orthorhombic)	8.19971	8.29623	11.80525	803.072	55.10
$CsPbBr_3$ NRs (intermediate)	8.20245	8.28058	11.85245	805.031	67.27

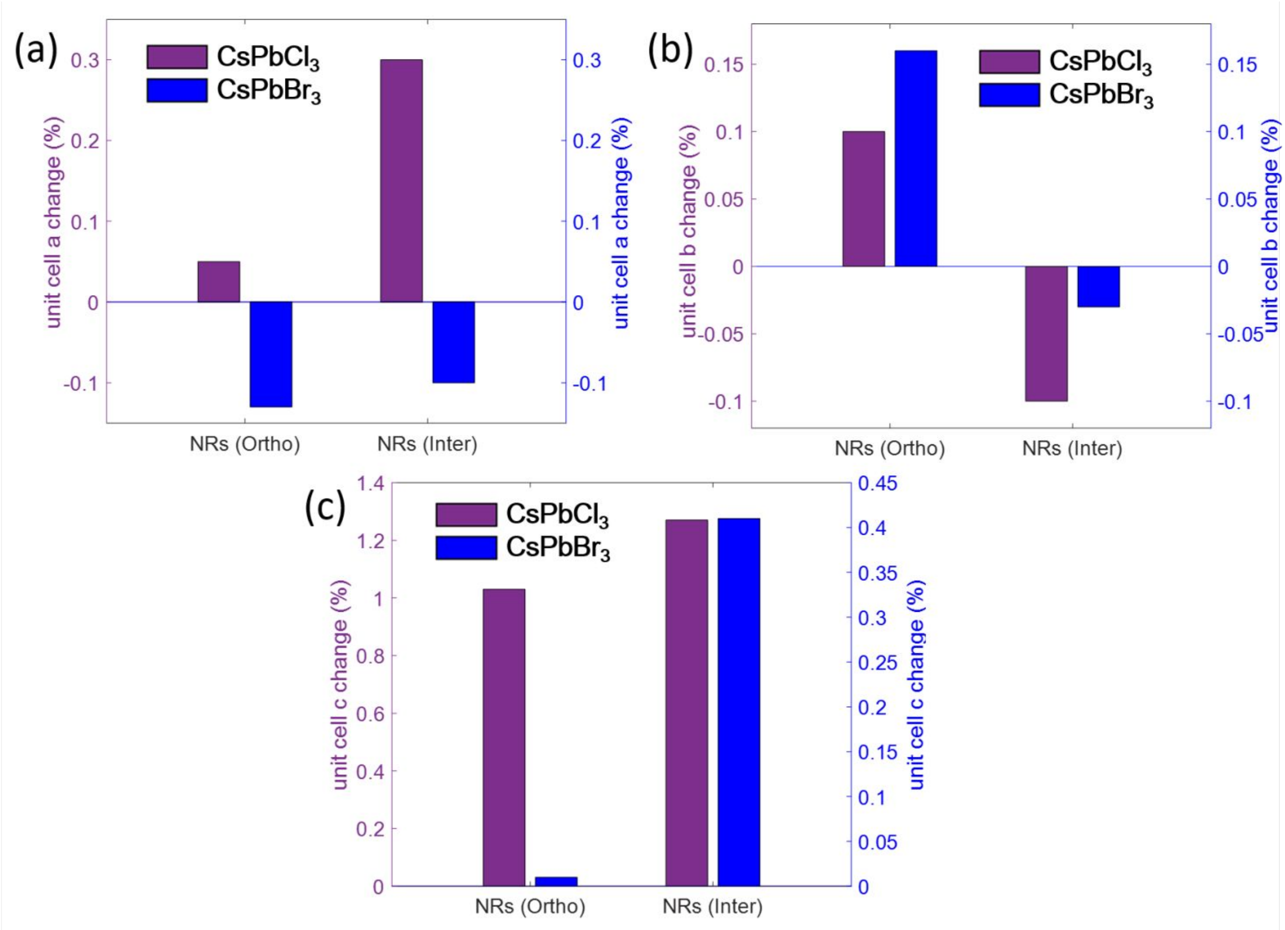

Figure S3. Fitted lattice parameter results of parent and Br-exchanged nanorods as compared to bulk materials in (a) a, (b) b, and (c) c axes.

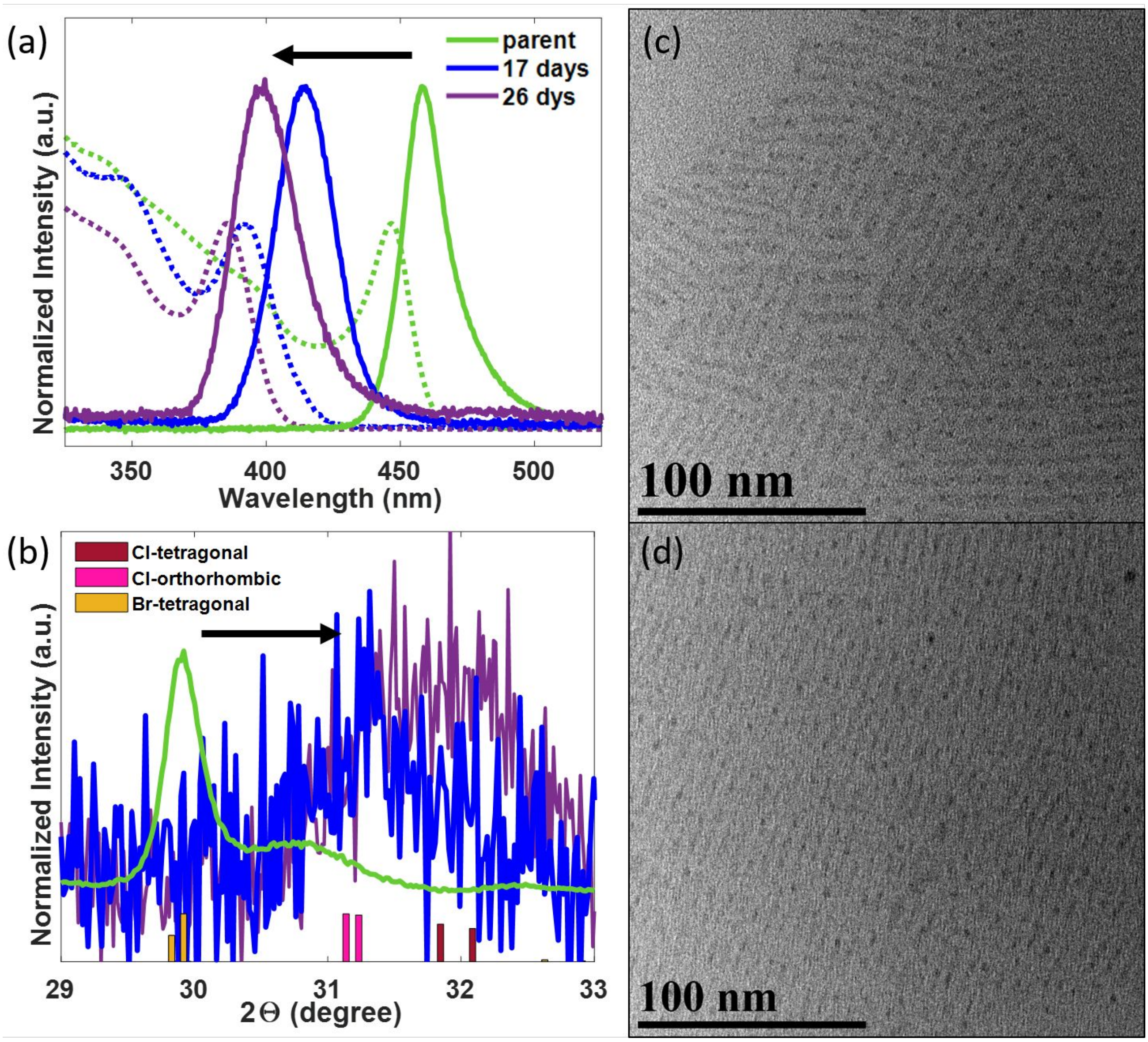

Figure S4. (a) UV-Vis spectra and (b) XRD patterns of parent tetragonal $CsPbBr_3$ nanorods and samples treated with $PbCl_2$ for 17 and 26 days. TEM images of (c) parent $CsPbBr_3$ nanorods and (d) samples treated for 26days. In (a), the absorption and PL spectra are displayed in dashed and solid lines, respectively.

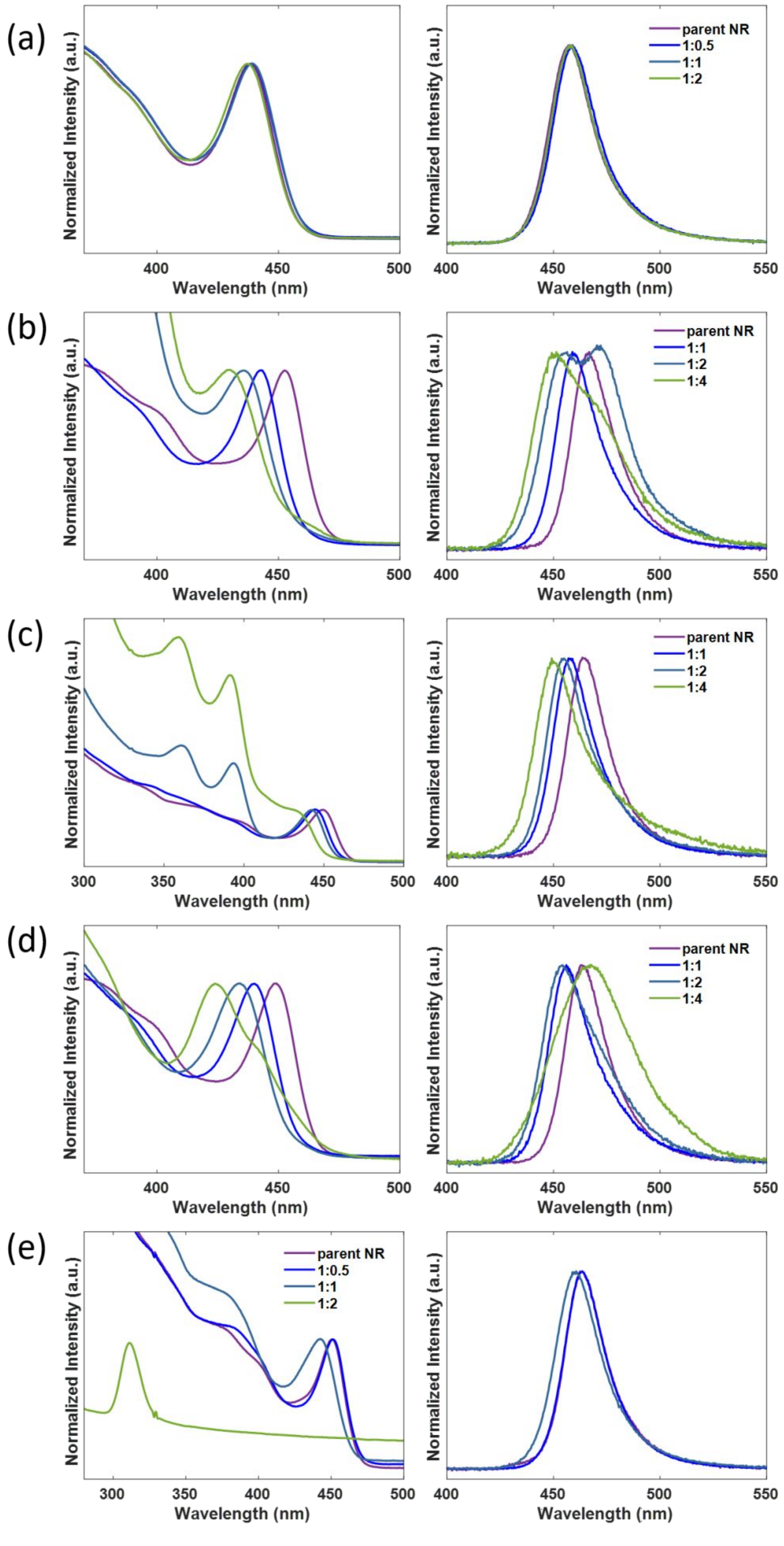

Figure S5. The absorption (left) and PL (right) spectra of samples with addition of varied molar ratios of (a) OA, (b) OAm, (c) Cs-OA, (d) Pb-OA, and (e) OAmHBr.

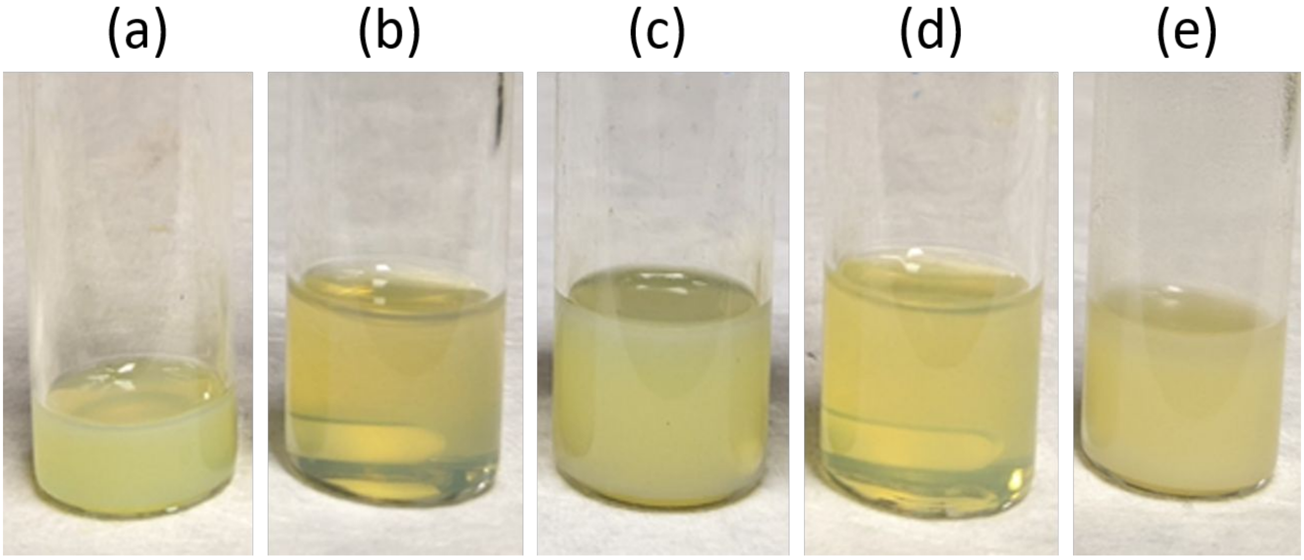

Figure S6. Photographs of (a) parent $CsPbBr_3$ nanorods and the samples treated with (b) OAm, (c) Cs-OA, (d) Pb-OA, and (e) OAmHBr.

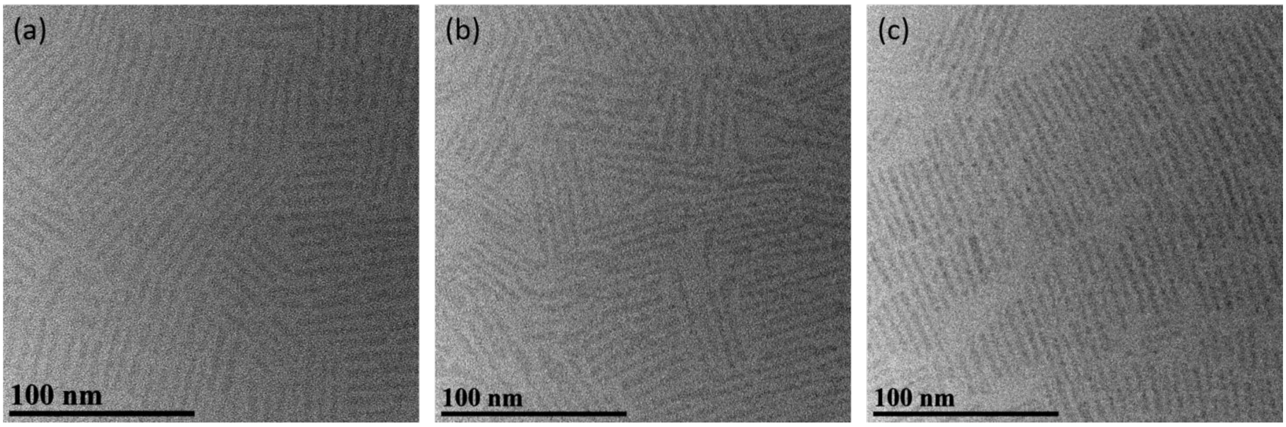

Figure S7. TEM images of (a) parent $CsPbBr_3$ nanorods and (b,c) samples with additional Pb-OA. The Pb molar ratios between nanorods and Pb-OA stock solutions are (b) 1:1 and (c) 1:4.

Table S3. Average sizes of nanorods before and after treatment with Pb-OA.*

Sample	parent NR	1:1	1:4
Diameter	4.88 ± 0.70	4.39 ± 0.64	3.91 ± 0.58
Length	42.27 ± 10.68	43.79 ± 8.49	40.19 ± 9.68

*units: nm

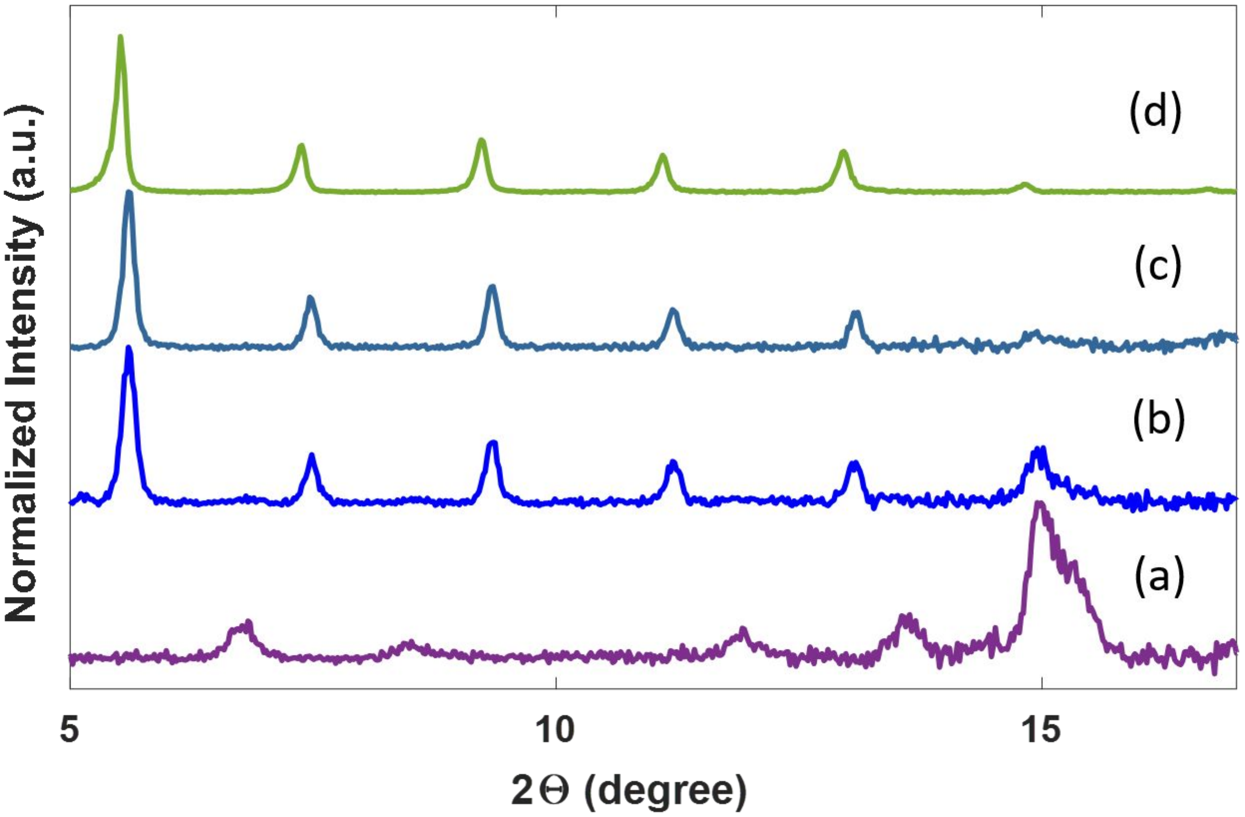

Figure S8. XRD patterns of (a) parent $CsPbBr_3$ nanorods and (b-d) samples with additional OAmHBr. The Br molar ratios between nanorod solutions and additional stocks are (b) 1:0.5 (c) 1:1, and (d) 1:2.

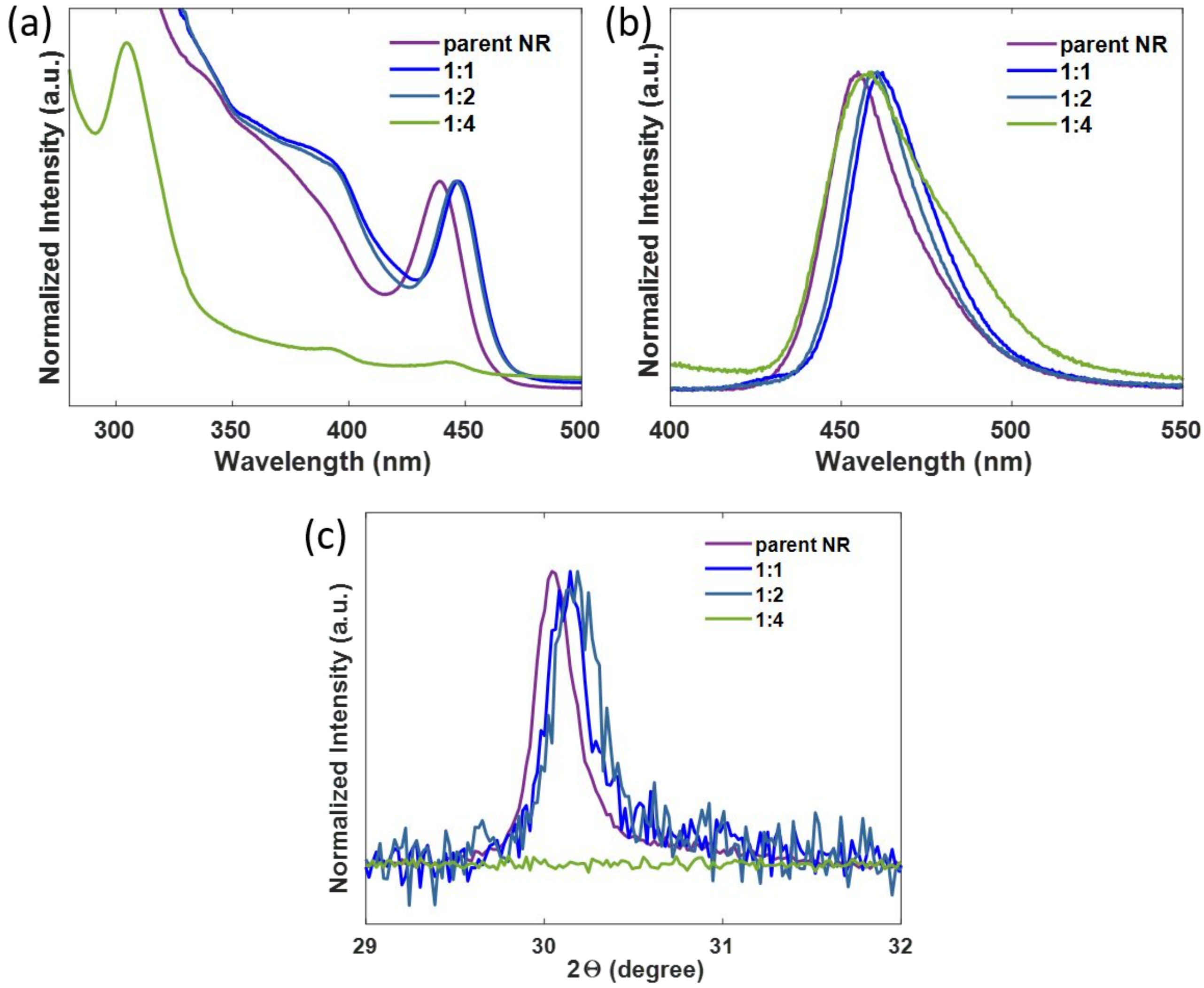

Figure S9. (a) Absorption, (b) PL spectra, and (c) XRD patterns of $CsPbBr_3$ nanorods treated with mixtures of Pb-OA and OAmHBr.

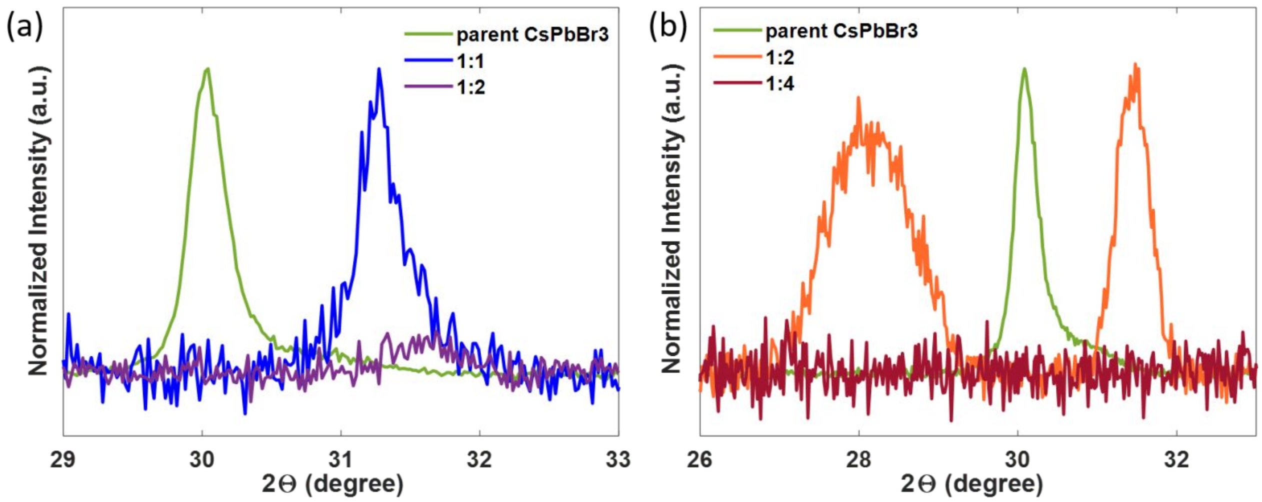

Figure S10. XRD patterns of $CsPbBr_3$ nanorods treated with (a) Bz-Cl and (b) Bz-I. In (b) the peak around 31.8^o is assigned to $Cs_4Pb(I/Br)_6$.

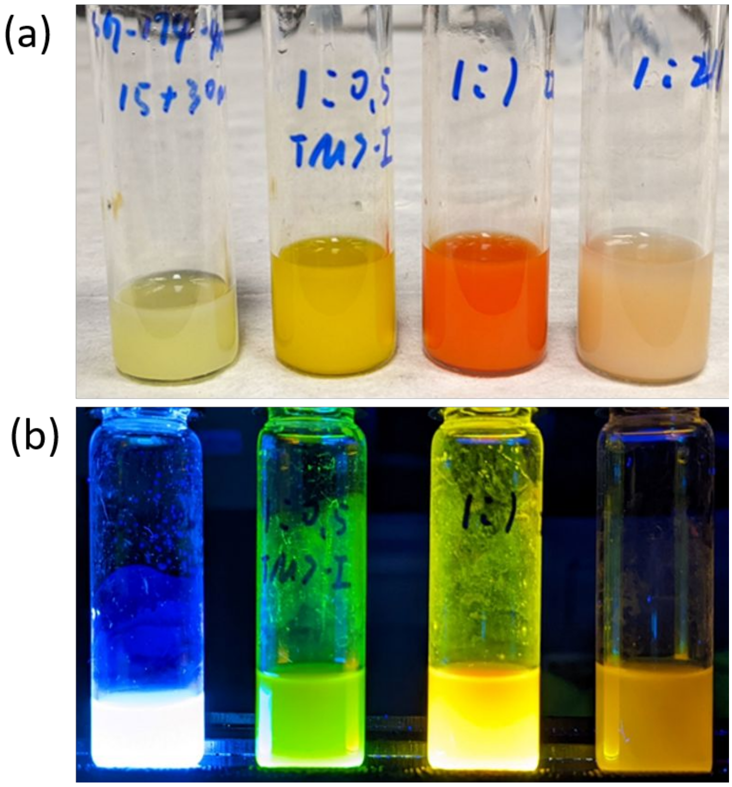

Figure S11. Photographs of $CsPbBr_3$ nanorods treated with varied concentrations of TMS-I illuminated with (a) room light and (b) UV light. The NRs became white cloudy and nearly non-florescent in a few minutes after the addition of x2 molar ratio of TMS-I (first vial on right).

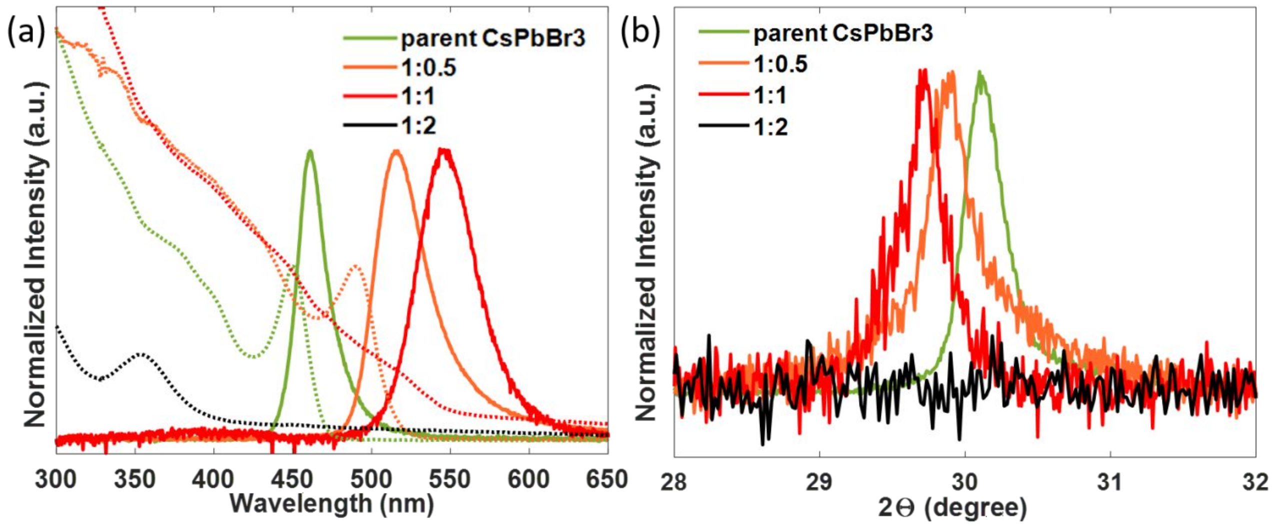

Figure S12. (a) UV-Vis spectra and (b) XRD patterns of $CsPbBr_3$ nanorods treated with varied amounts of TMS-I.

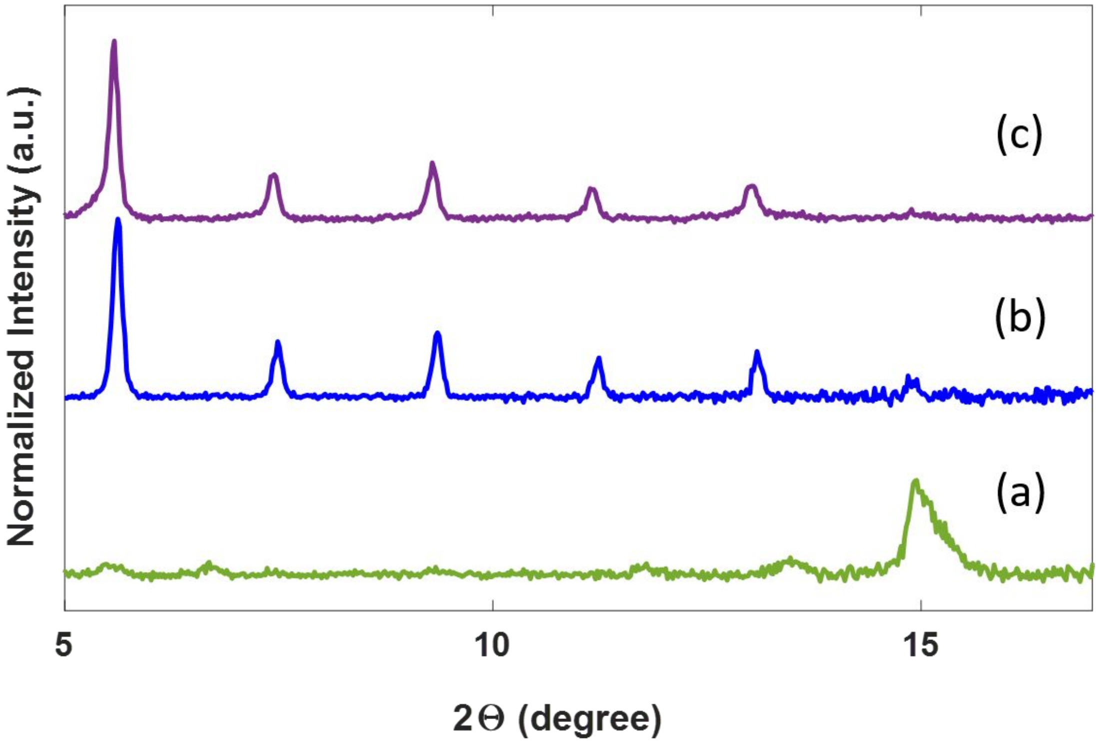

Figure S13. XRD patterns of (a) parent $CsPbBr_3$ nanorods and samples treated with (b) DHAmHBr and (c) TOAmHBr.
